# 

**Published:** 1896-03

**Authors:** 


### TABLE OF CONTENTS.

ORIGINAL COMMUNICATIONS. PAGE

Plantation of Teeth. By Louis Jack, D.D.S., Philadelphia . . . . . . . 133

Is Uric Acid an Important Factor in Dental Disease? By George D. B. Darby, Philadelphia . . . . . . . . . . . . . . . . . . . . . . . . 143

Dental Anæsthesia by Cataphoresis. By William H. Rollins, Boston, Mass. . . . . . . . . . . . . . . . . . . . . . . . . . . . . 151

REPORTS OF SOCIETY MEETINGS.

American Dental Association . . . . . . . . . . . . . . . . . . . . . 153

Academy of Stomatology . . . . . . . . . . . . . . . . . . . . . . . 169

New York Odontological Society . . . . . . . . . . . . . . . . . . . 175

EDITORIAL.

The Parasite in Science . . . . . . . . . . . . . . . . . . . . . . . 190

Dr. Williams's Criticism . . . . . . . . . . . . . . . . . . . . . . 192

Dr. W. H. Dwinelle . . . . . . . . . . . . . . . . . . . . . . . . . 193

FOREIGN CORRESPONDENCE.

Reply to the January Editorial . . . . . . . . . . . . . . . . . . . 194

CURRENT NEWS.

Woman's Dental Association . . . . . . . . . . . . . . . . . . . . . 196

